# A MIV-150/Zinc Acetate Gel Inhibits SHIV-RT Infection in Macaque Vaginal Explants

**DOI:** 10.1371/journal.pone.0108109

**Published:** 2014-09-26

**Authors:** Patrick Barnable, Giulia Calenda, Louise Ouattara, Agegnehu Gettie, James Blanchard, Ninochka Jean-Pierre, Larisa Kizima, Aixa Rodríguez, Ciby Abraham, Radhika Menon, Samantha Seidor, Michael L. Cooney, Kevin D. Roberts, Rhoda Sperling, Michael Piatak, Jeffrey D. Lifson, Jose A. Fernandez-Romero, Thomas M. Zydowsky, Melissa Robbiani, Natalia Teleshova

**Affiliations:** 1 Population Council, New York, New York, United States of America; 2 Aaron Diamond AIDS Research Center, Rockefeller University, New York, New York, United States of America; 3 Tulane National Primate Research Center, Tulane University, Covington, Louisiana, United States of America; 4 Icahn School of Medicine at Mount Sinai, New York, New York, United States of America; 5 AIDS and Cancer Virus Program, SAIC-Frederick, Inc., Frederick National Laboratory for Cancer Research, Frederick, Maryland, United States of America; University of California, San Francisco, United States of America

## Abstract

To extend our observations that single or repeated application of a gel containing the NNRTI MIV-150 (M) and zinc acetate dihydrate (ZA) in carrageenan (CG) (MZC) inhibits vaginal transmission of simian/human immunodeficiency virus (SHIV)-RT in macaques, we evaluated safety and anti-SHIV-RT activity of MZC and related gel formulations *ex vivo* in macaque mucosal explants. In addition, safety was further evaluated in human ectocervical explants. The gels did not induce mucosal toxicity. A single *ex vivo* exposure to diluted MZC (1∶30, 1∶100) and MC (1∶30, the only dilution tested), but not to ZC gel, up to 4 days prior to viral challenge, significantly inhibited SHIV-RT infection in macaque vaginal mucosa. MZC's activity was not affected by seminal plasma. The antiviral activity of unformulated MIV-150 was not enhanced in the presence of ZA, suggesting that the antiviral activity of MZC was mediated predominantly by MIV-150. *In vivo* administration of MZC and CG significantly inhibited *ex vivo* SHIV-RT infection (51–62% inhibition relative to baselines) of vaginal (but not cervical) mucosa collected 24 h post last gel exposure, indicating barrier effect of CG. Although the inhibitory effect of MZC (65–74%) did not significantly differ from CG (32–45%), it was within the range of protection (∼75%) against vaginal SHIV-RT challenge 24 h after gel dosing. Overall, the data suggest that evaluation of candidate microbicides in macaque explants can inform macaque efficacy and clinical studies design. The data support advancing MZC gel for clinical evaluation.

## Introduction

Considering that sexual intercourse is the main route of HIV acquisition, products that block sexual HIV-1 transmission are urgently needed. The success of the CAPRISA 004 clinical trial, in which vaginal application of a 1% tenofovir gel reduced HIV-1 acquisition by 39% overall and by 54% in high adherers [1], emphasizes the promise for anti-HIV microbicide development.

The Population Council's (PC's) lead microbicide gel (MZC) containing 50 µM of the NNRTI MIV-150 and 14 mM zinc acetate dihydrate (ZA) in carrageenan (CG) protects Depo-Provera-treated macaques against a single vaginal simian/human immunodeficiency virus (SHIV)-RT challenge completely for up to 8 h and protects ∼75% of macaques when challenged 24 h after gel dosing (original and modified gels optimized for use in human) [2]–[4]. Although not as effective as MZC, repeated application of ZC significantly protected macaques [2], [5], but this was reduced when ZC was only administered once [4]. Importantly, the efficacy of MC was improved by the addition of ZA [2]. ZA could contribute to the antiviral activity of MZC through zinc targeting the reverse transcriptase (RT), involving sites not targeted by traditional RTIs (Mizenina et al., in preparation) [6] and induction of immune changes (e.g., cytokines/chemokines/other innate factors) [7]–[10] that can also influence infection.

This study was designed to determine (i) e*x vivo* safety of MZC in macaque and human explants, (ii) anti-SHIV-RT activity of MZC and related gel formulations *ex vivo* in macaque mucosal explants, and (iii) pharmacodynamics (PD) of MZC in macaque mucosal tissues after *in vivo* gel application to support advancing this formulation for clinical testing.

## Materials and Methods

### Ethics statement

Adult female Chinese and Indian rhesus macaques (*Macaca mulatta*) were housed and cared for in compliance with the regulations under the Animal Welfare Act [11], the Guide for the Care and Use of Laboratory Animals [12], at Tulane National Primate Research Center (TNPRC; Covington, LA). All studies were approved by the Animal Care and Use Committee of the TNPRC (OLAW assurance #A4499-01). Animals were monitored continuously by veterinarians to ensure their welfare. Animals in this study were socially housed unless restricted by study design. Housing restrictions were scientifically justified and approved by the IACUC as part of protocol review. Animals were housed indoors in climate controlled conditions with a 12/12 light/dark cycle. Animals in this study were fed commercially prepared monkey chow twice daily. Supplemental foods were provided in the form of fruit, vegetables, and foraging treats as part of the TNPRC environmental enrichment program. Water was available at all times through an automatic watering system. The TNPRC environmental enrichment program is reviewed and approved by the IACUC semiannually. Extensive efforts are made to find compatible pairs for every study group, with additional environmental enrichment of housing space through a variety of food supplements and physical complexity of the environment. A team of 11 behavioral scientists monitors the wellbeing of the animals and provides direct support to minimize stress during the study period. All biopsy procedures were performed by Board Certified veterinarians (American College of Laboratory Animal Medicine). Veterinarians at the TNPRC Division of Veterinary Medicine have established procedures to minimize pain and distress through several means. Anesthesia was administered prior to and during all procedures, and analgesics were provided afterwards as previously described [2], [13]. Seven macaques became sick over the course of this study and were euthanized. Leftover necropsy tissues derived from 14 additional animals (8 sick and 6 healthy) were available from separate studies. These animals were euthanized using methods consistent with recommendations of the American Veterinary Medical Association (AVMA) Guidelines for Euthanasia. The animal is anesthetized with tiletamine/zolazepam (8 mg/kg IM) and given buprenorphine (0.01 mg/kg IM) followed by an overdose of pentobarbital sodium. Death is confirmed by auscultation of the heart and pupillary dilation. TNPRC is accredited by the Association for Assessment and Accreditation of Laboratory Animal Care (AAALAC#000594).

### Macaques

Naïve, SIV infected, SHIV and SHIV-RT exposed/uninfected and infected recycled animals were utilized. The plasma viral load of the infected animals used for *ex vivo* gel activity testing and in PD studies ranged from undetectable (lower limit of quantification  =  30 copies/ml) to 50 copies/ml by quantitative RT-PCR for SIV *gag* [14], [15]. Animals used for vaginal biopsy collection in *ex vivo* gel safety and activity testing experiments ranged in age from 4–21 years old and their weights ranged from 4.4–12.15 kg. Animals used in PD studies ranged in age from 5–15 years old and their weights ranged from 5.65–10.55 kg.

### Gels and active pharmaceutical ingredients (APIs)

The gel formulation attributes are summarized in Table 1. The original gels contained 95λ:5κ CG (lot PDR98-15) that is no longer available from the manufacturer, whereas the modified gels contained 60λ:40κ CG (lot RERK-4137) which is available. The design of modified gels was adapted for human use by changing 1% DMSO to 2% propylene glycol (PG) as the co-solvent and adjusting buffers [3]. Regardless of the co-solvent and other components, the gels likely contained a mixture of soluble and insoluble MIV-150. However, insoluble MIV-150 will dissolve as the soluble MIV-150 is absorbed into tissues, thereby keeping the amount of soluble MIV-150 constant. In the final manufacturing step, the pH of the gels was adjusted to 6.8±0.2, so any change in pH due to the addition of ZA was neutralized. As described in [2], MIV-150 was developed by Medivir AB (Sweden) and licensed to the PC. Unformulated MIV-150 and ZA were used by diluting stock solutions of 27 mM (MIV-150; in DMSO) and 14 mM (ZA; in sodium acetate buffer, pH adjusted to 6.2 with glacial acetic acid).

**Table 1:** Formulation attributes.

	Gels	CG (% w/w)	λ:κ CG (% of total CG)	ZA (% w/w, mM)	MIV-150 (% w/w, μM)	Buffer	Preservative	API solvent	Gel lots
**Original**	CG	3.0	95:5	-	-	PBS	0.2% MP	-	120118A515MR
	MZC	3.0	95:5	0.3,14	0.00184, 50	-	0.2% MP	DMSO	120119A1005MR
								-	110204A525ML
**Modified**	CG	3.4	60:40	-	-	C_2_H_3_NaO_2_	0.2% MP	PG	110512A525MR
								PG	120111525ML
								PG	110920A525
								PG	110218A1005ML
	MZC	3.1	60:40	0.3, 14	0.00184, 50	C_2_H_3_NaO_2_	0.2% MP	PG	110516A1005MR
								PG	120926A1005ML
								PG	110921A1005ML
	ZC	3.1	60:40	0.3, 14	-	C_2_H_3_NaO_2_	0.2% MP	-	110211A707ML
								PG	110609A707MR
	MC	3.4	60:40	-	0.00184, 50	C_2_H_3_NaO_2_	0.2% MP	PG	110513A815ML

Abbreviations: CG, carrageenan; ZA, zinc acetate dihydrate; DMSO, dimethyl sulfoxide; PG, propylene glycol; C_2_H_3_NaO_2_, sodium acetate; MP, methylparaben.

### Macaque vaginal tissues for testing gel safety and anti-SHIVRT activity *ex vivo*

Vaginal mucosa (n = 2 biopsies; or necropsy tissues) was obtained from untreated naïve and recycled macaques. Biopsies were collected using 3×5 mm biopsy forceps. To determine the activity of MZC in tissues from Depo-Provera (Depo) treated animals, a single 30 mg dose of Depo was given to animals by intramuscular injection and vaginal biopsies were collected 5 weeks (wks) later. Tissues were placed in complete L-15 (cL-15) medium (L-15 (HyClone Laboratories, Inc., Logan, UT) supplemented with 10% FBS (Gibco, Life Technologies, Grand Island, NY), 100 U/ml penicillin - 100 µg/ml streptomycin (Cellgro Mediatech, Manassas, VA) and transported to our laboratories at the PC on ice overnight.

### Human ectocervical tissues for testing gel safety

Human ectocervical tissues without gross pathological changes from women undergoing routine hysterectomy were received from the National Disease Research Interchange (NDRI, Philadelphia, PA) and from Icahn School of Medicine at Mount Sinai (New York, NY). No patient identifiers were provided. The study was conducted under IRB approved protocol (Program for the Protection of Human Subjects, Icahn School of Medicine at Mount Sinai; #10-1213 0001 01 OB). Participants provided written informed consent. The consent procedure was approved by the IRB. Tissues were processed fresh or transported on ice overnight in RPMI medium.

### *In vivo* treatments and sample collection for PD studies

**Figure 1:**
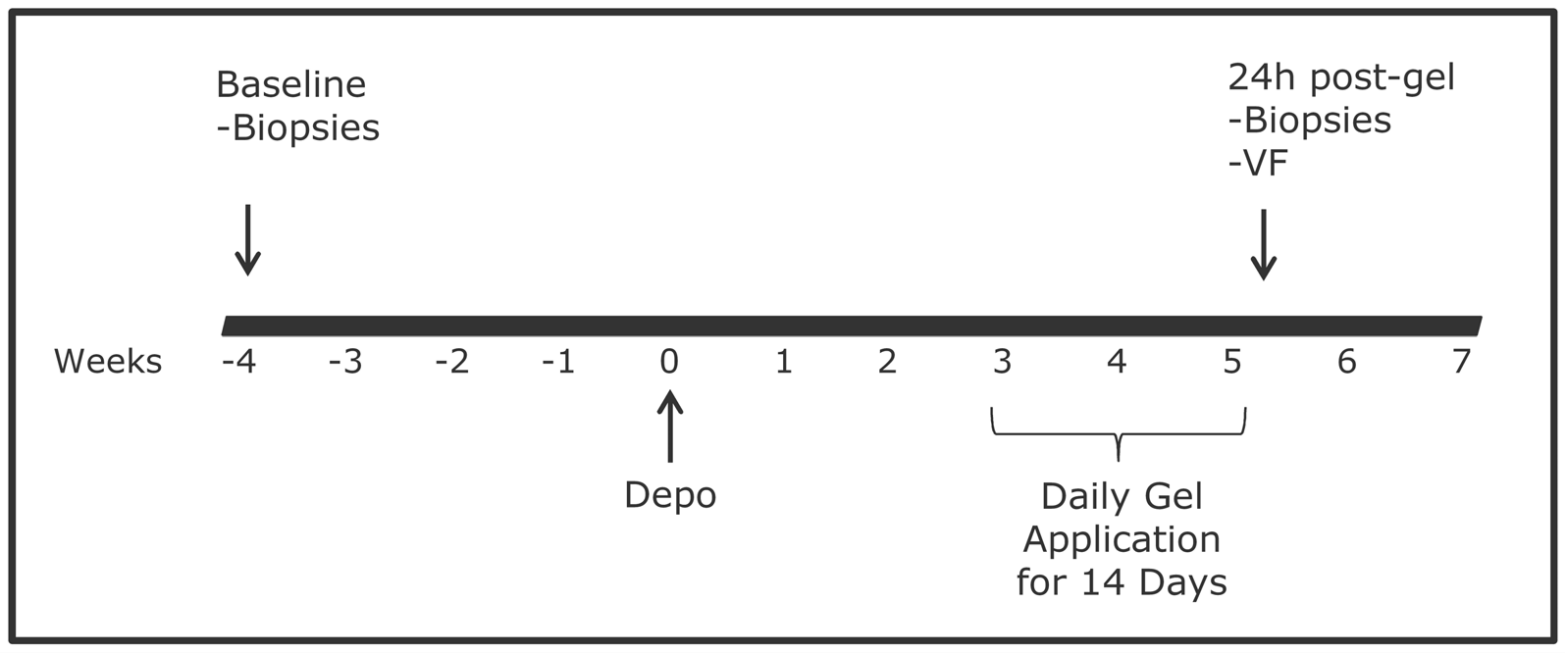
Study design schematic. Macaques were treated with 2 mL of MZC (n = 9) or CG gel (n = 8) intravaginally daily for 14d. Vaginal biopsies were collected from all macaques at the baseline (4 wks before Depo treatment) and 24 h after the last gel exposure to determine PD of MZC in tissues. Ectocervical biopsies were collected from 5 macaques in both groups. VF was collected just before collection of biopsies to assess PK of MIV-150.

The design of PD studies is shown in Fig. 1. Naïve and recycled animals were given Depo treatment as described above. Three wks after Depo treatment, 2 ml of the MZC gel or the corresponding CG placebo gel were applied intravaginally daily for 2 wks to mirror the conditions of the efficacy studies [2], [3]. Specimens (vaginal fluids (VF); vaginal or vaginal and ectocervical tissues (two biopsies each)) were collected at baseline (4 wks prior to Depo treatment) and 24 h post last gel administration. VF were collected using foam swabs [16]. Both original (n = 4 and n = 3 animals in MZC and CG groups, respectively) and modified (n = 5 animals in MZC and CG groups each) gels were tested. Swabs were transported to our laboratories at the PC on ice overnight and processed as previously described [16], [17]. Tissues were placed in cL-15 medium and immediately processed for infection (below).

### Viral stocks

SHIV-RT was generated from the original stock provided by Disa Böttiger (Medivir AB, Sweden) [18] using PHA/IL-2-activated macaque PBMCs and titered in CEMx174 cells before use as previously described [2], [17]. The same viral stock was used for *ex vivo* tissue challenge at the baseline and post gel exposure in PD studies.

### Histology

Histological analysis was performed on macaque vaginal and human ectocervical mucosae. Tissues were washed in PBS and excess submucosa was trimmed. Then tissues were processed, adapting the protocol described for polarized explant culture [19]. Briefly, 5×5×3 mm explants cut using 5 mm diameter Acu-Punches (Acuderm, Fort Lauderdale, FL) were inserted in a 3 mm hole in a 3 µm pore polyester membrane transwell insert (Costar, Corning, NY) with the mucosal side facing upwards. The positioned tissues were sealed with Matrigel basement membrane matrix (BD Biosciences, Franklin Lakes, NJ), and cDMEM (DMEM (Cellgro Mediatech) containing 10% FBS (for use in macaque tissue) (Gibco), or 10% AB serum (for use in human tissue) (Sigma Aldrich, St. Louis, MO), 100 U/ml penicillin - 100 µg/ml streptomycin (Cellgro Mediatech), 100 µM MEM non-essential amino acids (Irvine Scientific, Santa Ana, CA)) was placed in the basolateral compartment. The mucosal side of the culture was exposed overnight (∼18 h) to neat or 1∶10 diluted gel formulations. A single explant was set up for each condition. After washing in PBS, tissues were placed in 10% buffered formalin (Richard-Allan Scientific, Kalamazoo, MI) for at least 24 h and submitted to the Laboratory of Comparative Pathology (Cornell University, New York, NY) for paraffin embedding, cutting and Hematoxilin and Eosin (H&E) staining. Tissue sections (5 µm) were examined at the Bioimaging Resource Center of The Rockefeller University using a Zeiss Axioplan 2 widefield microscope (Carl Zeiss Microscopy, Thornwood, NY). Brightfield pictures were taken at a 10× magnification.

### Tissue viability

5×5×3 mm or 3×3×3 mm polarized tissue cultures of macaque vaginal and human ectocervical explants (inserted in 3 mm or 2 mm holes in the transwell inserts, respectively; single explant/condition) were set up as described in the “Histology” section. To test potential gel toxicity in an immersion culture model, 3×3×3 mm explants were set up (2–4 explants/condition) in a 96-well plate. Tissues were incubated with the gels (neat or diluted) or medium control overnight (∼18 h) and toxicity measured as described [17], [20].

### Activity of APIs and gels against e*x vivo* SHIV-RT challenge

Transported macaque vaginal tissue was processed with minor modifications to the published protocol [20]. All viral challenges were performed in an immersion model. Briefly, 3×3×3 mm explants were stimulated with 5 µg/ml PHA (Sigma Aldrich) and 100 U/ml IL-2 (Roche Applied Science, Indianapolis, IN; or NCI BRB Preclinical Repository, Frederick, MD) in cDMEM for 48 h. Then, tissues were challenged with SHIV-RT (10^4^ TCID_50_ per explant) overnight (∼18 h) in the presence of gels (2–4 explants/condition), washed (x4) and cultured in the presence of 100 U/ml IL-2 for 14d. Tissue culture supernatants were collected at days 0 (d0; post last wash), 3, 7, 11 and 14 of culture, and infection was monitored by RETRO-TEK SIV p27 Antigen ELISA kit (ZeptoMetrix, Buffalo, NY). To test if MZC retained its activity in the presence of seminal plasma (SP), activated explants were challenged with SHIV-RT in the presence of diluted gel and 12.5% of pooled human SP (Lee Biosolutions Inc., St. Louis, MO). Pooled semen was centrifuged at 500x*g* for 10 minutes (min), 4°C for the separation of SP. SP was collected, aliquoted and stored at −20°C until use.

To determine if the APIs maintained any residual activity after washout, tissues were incubated with either unformulated MIV-150, ZA, or both in combination, or with the gels overnight (∼18 h) in the presence of 5 µg/ml PHA and 100 U/ml IL-2 in cDMEM (2–4 explants/condition). Then tissues were washed (x4) and cultured in the presence of PHA/IL-2 in the same tissue culture plate. 24 h or 4d post API or gel washout, tissues were challenged with SHIV-RT as described above and cultured in the presence of IL-2 for 14d. In the 4d post gel exposure challenge experiments, tissues were additionally washed (x2) after ∼48 h of PHA/IL2 stimulation to eliminate PHA. In separate experiments to address whether residual MIV-150 bound to the tissue culture plate contributed to the observed activity, tissues were transferred to a new plate after gel exposure and washout. As SHIV exposed animals were included in the studies, we measured basal p27 production in cultured vaginal tissues from these animals. No basal p27 production was detected (at least 40 experiments, not shown). Based on these results and due to the limited amount of biopsy tissues, the no-SHIV-RT-challenge control condition was not included in the studies.

### Tissue PD studies

Anti-SHIV-RT activity of tissue-associated MIV-150 was determined in biopsies collected 24 h post last MZC and CG placebo gel application (vs. biopsies collected at the baselines) (Fig. 1). Biopsies (n = 2 vaginal and ectocervical) were collected using 3×5 mm and 3×4.5 mm forceps for vaginal and ectocervical tissue, respectively. Tissues were processed for SHIV-RT challenge in an immersion model as described [17], [20] within an hour of collection. Non-stimulated explants (3–6 vaginal explants and 2–5 ectocervical) were challenged with 10^5^ TCID_50_ SHIV-RT per explant in the presence of IL-2 (100 U/ml) (no PHA) and cultured for 14d as described above. This challenge dose was found to reproducibly infect tissues that were not pre-stimulated with PHA/IL-2 [17]. ∼18 h after infection, tissues were washed and cultured in cDMEM containing IL-2 for 13 or 14d. Supernatants were collected at 0 (after washes), 3, 7, 10/11 and 13/14d of culture. Infection was monitored as described above.

### SIV *gag* qPCR

DNA was extracted from tissues using the DNeasy Blood and Tissue Kit (Qiagen, Germantown, MD) after 3 or 7d of culture post SHIV-RT challenge for the quantification of viral DNA. qPCR was performed using the ABsolute Blue QPCR SYBR Green Low ROX mix (Thermo Scientific, Waltham, MA) and the Viia 7 real time PCR system (Applied Biosystems, Carlsbad, CA). Changes in SIV *gag* expression were analyzed by the comparative crossing threshold (*C*_t_) method (2^−ΔΔ*C*t^ method) [21] as previously described [20]. Primers for SIV were 5′-GGTTGCACCCCCTATGACAT-3′ (SIV667gag Fwd) and 5′-TGCATAGCCGCTTGATGGT-3′ (SIV731gag Rev); primers for the reference gene albumin were 5′-ATTTTCAGCTTCGCGTCTTTTG-3′ (RhAlbF) and 5′-TTCTCGCTTACTGGCGTTTTCT-3′ (RhAlbRev) [20].

### RIA for MIV-150 detection

MIV-150 was detected in VF by radioimmunoassay (RIA) as previously described [2], [17]. The MIV-150 concentration in the samples was calculated using a curve fitting procedure (logistic 4-parameter model).

### Statistics

#### Tissue viability analysis

Analysis of tissue viability (MTT assay) in the presence of gels was done using a log-normal generalized linear mixed model predicting the weight-normalized OD_570_ of each replicate. A random intercept was included by animal ID. The significance was determined by the Type 3 *F*-test for overall treatment effect. Significant pairwise comparisons to medium were determined by t tests with Dunnett's adjustment.

#### Activity of gels against *ex vivo* SHIV-RT challenge

Analysis of gel activity against *ex vivo* SHIV-RT challenge was done using a log-normal generalized linear mixed model with the individual replicate data. For CUM and SOFT analyses [22], [23], p27 concentrations of individual replicate values ≥ LLOQ were assumed log-normal. Any value <LLOQ (62.5 pg/ml) at 3-14d of culture was set to

62.5^1√2^ = 18.616, a common substitution for log-normal data. CUM from 3-14d for replicates below LLOQ corresponds to 74.46 pg/ml [17]. A random intercept was included by animal ID. For experiments with recycled animals, a random effect of biopsy date was included grouped by animal ID.

#### Tissue PD studies

CUM and SOFT analyses were done as described above. Comparisons of CUM and SOFT p27 endpoints were performed using a log-normal generalized linear mixed model with the individual replicate data. Because in the PD study baseline infection was monitored over 14d of culture but post gel infection was monitored over 13d of culture, a linear extrapolation was used to predict post gel p27 concentrations at day 14. The treatment (MIV-150 vs. placebo), type of gel (original vs. modified), biopsy time (baseline vs. post gel exposure), and all interactions were used a predictors. Time was also included as a random effect grouped by a random subject of animal ID. To examine vaginal vs. ectocervical tissue, the treatment, biopsy tissue type (ectocervical vs. vaginal), biopsy time, and all interactions were used a predictors. Time was also included as a random effect grouped by a random subject of animal ID. Overall significance was determined by the Type 3 *F*-test, and pairwise comparisons were made with Tukey-adjusted *t* tests. In all cases, macaque age (animal age spanned 10 years) and interactions with age were not significant and were excluded from the final models.

All analyses were performed with SAS V9.4, SAS/STAT V13.2 and α = 0.05. Significant *p*-values of <0.05 (*), <0.01 (**) and <0.001 (***) are indicated.

## Results

### MZC does not induce toxic changes in macaque vaginal and human ectocervical mucosa

**Figure 2:**
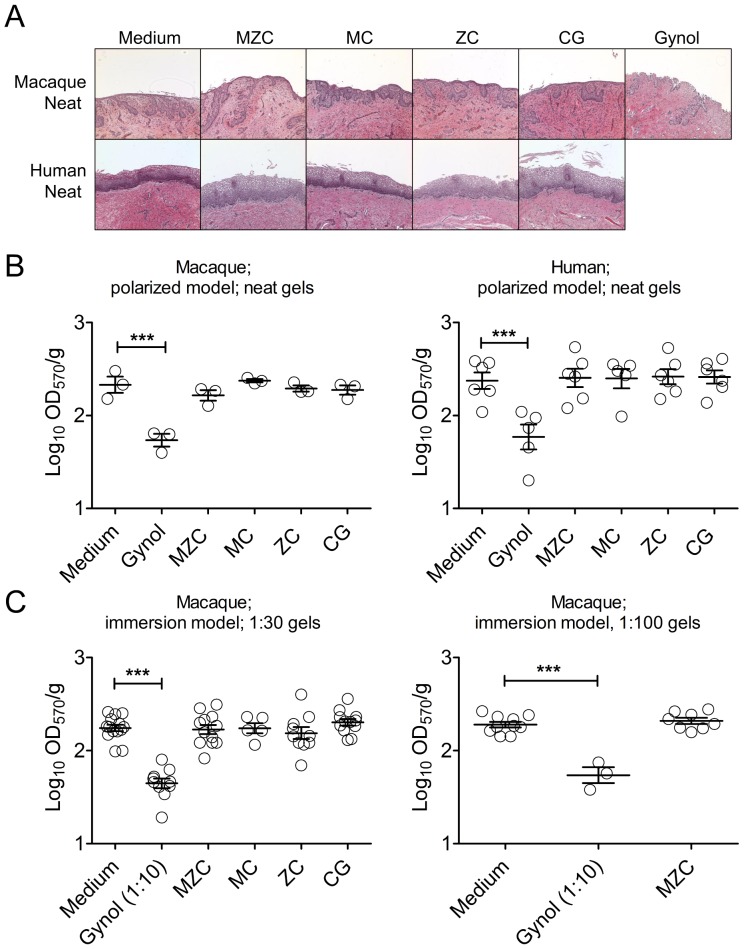
MZC is not toxic in macaque vaginal and human ectocervical explants. (A) Polarized macaque vaginal and human ectocervical explants were cultured for ∼18 h in the presence of neat modified gels, applied on the epithelium. For histological evaluation, after exposure to MZC, MC, and ZC, or control conditions (medium, CG, Gynol), tissues were washed, paraffin-embedded, and stained with H&E. Results representative of 4–6 (macaque) and 1–2 (human) experiments are shown at 10× magnification. (B) For determination of tissue viability polarized macaque vaginal and human ectocervical explants (single tissues) were incubated with neat modified MZC, MC, ZC (vs. Medium, CG and Gynol controls) applied on the epithelium for ∼18 h. Each symbol indicates a donor and the Mean±SEM of the log_10_(OD_570_/g) for each condition is shown. (C) Macaque vaginal explants (2–4 per condition) were immersed in culture media with diluted (1∶30 or 1∶100) modified gels (vs. Medium and 1∶10 diluted Gynol control) for ∼18 h. Following incubation with the gels, tissue viability was determined using MTT assay. Each symbol indicates a donor and the Mean±SEM of the log_10_(OD_570_/g) for each condition is shown.

We performed histological evaluation (H&E staining) and viability testing (MTT assay) of macaque vaginal and human ectocervical tissues to assess the safety of modified MZC and related gels. No histopathological changes were detected in the macaque and human mucosa after application of neat test gels on the epithelial surface of the explants for 18 h (Fig. 2A). Similarly, no histopathological changes were observed when 1∶10 diluted (to mimic gel dilution *in vivo*) MZC, MC, CG were applied on human ectocervical mucosa (3 experiments, not shown). The tissue viability (MTT assay) was not decreased after exposure to MZC, ZC, MC or CG relative to the medium control and was significantly decreased after exposure to Gynol (Fig. 2B). The tissue viability was also determined in the immersion culture model in order to ensure that the antiviral activity testing was performed using non-toxic gel dilutions. We previously determined that ZC gel (original formulation) significantly decreases tissue viability at 1∶10 (42% viability of control; p-value = 0.0001) and at 1∶20 dilutions (64% viability of control; p-value = 0.0435), but not at 1∶30 dilution (84% viability of control; p-value = 0.3474) (3–10 experiments; not shown). Based on this, macaque vaginal explants were immersed in 1∶30 and 1∶100 diluted modified MZC and related gels for ∼18 h and processed for the MTT assay. No significant changes in tissue viability were detected after immersion in diluted gels (Fig. 2C).

### MZC protects against *ex vivo* SHIV-RT infection in macaque vaginal explants and this is not affected by SP

**Figure 3:**
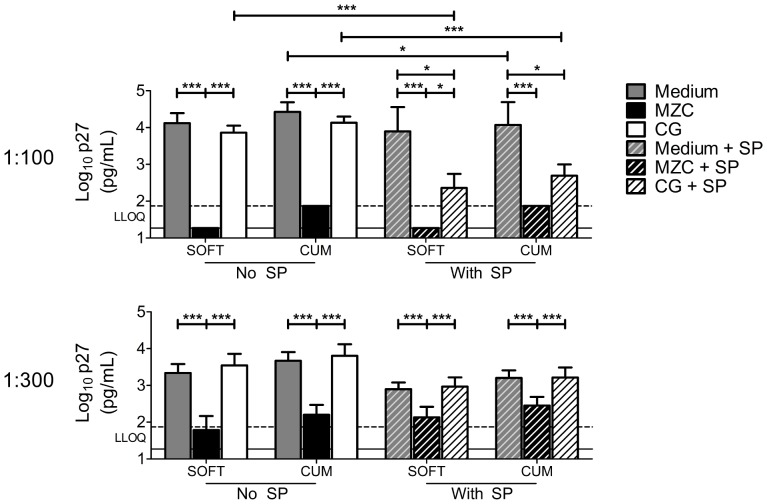
MZC and MC inhibit SHIV-RT infection in macaque vaginal explants. Explants stimulated with PHA/IL-2 for 48 h were challenged with SHIV-RT (10^4^ TCID_50_/explant; 2–4 explants/condition) in the presence of modified MZC diluted 1∶100 or 1∶300 with or without 12.5% human SP. Explants challenged in medium alone or in the presence of CG (± SP) served as controls. After ∼18 h, the tissues were washed and cultured for 14d in the presence of IL-2. SIV p27 release was measured at 0, 3, 7, 11, 14d of culture by ELISA. Shown are log_10_-transformed p27 SOFT and CUM analyses (Mean±SEM) (d3-14 of culture). Summary of n = 3–8 (top panel) and n = 5–8 (bottom panel) experiments are shown. The LLOQ of the assay are denoted for both SOFT (solid line) and CUM (dotted line).

Macaque vaginal explants from untreated animals were challenged with 10^4^ TCID_50_ of SHIV-RT in the presence of 1∶100 or 1∶300 diluted modified MZC and SP (12.5%) for ∼18 h. To allow reproducible infection, explants were stimulated with PHA/IL-2 prior to challenge. MZC at both 1∶100 and 1∶300 dilution in the absence of SP provided strong inhibition of infection relative to untreated medium and CG placebo controls (Fig. 3). Overall, tissue infection in the presence of non-toxic 12.5% SP (MTT data not shown) was lower than control infection (SOFT/CUM p-values = 0.0273/0.0142; combined Medium vs. Medium + SP data (Fig. 3)). Anti-SHIV-RT activity of MZC was not changed in the presence of SP (Fig. 3). 1∶100 (but not 1∶300) diluted CG + SP inhibited infection relative to medium + SP control and CG.

### MZC provides post washout activity against *ex vivo* SHIV-RT infection in macaque vaginal explants

**Figure 4:**
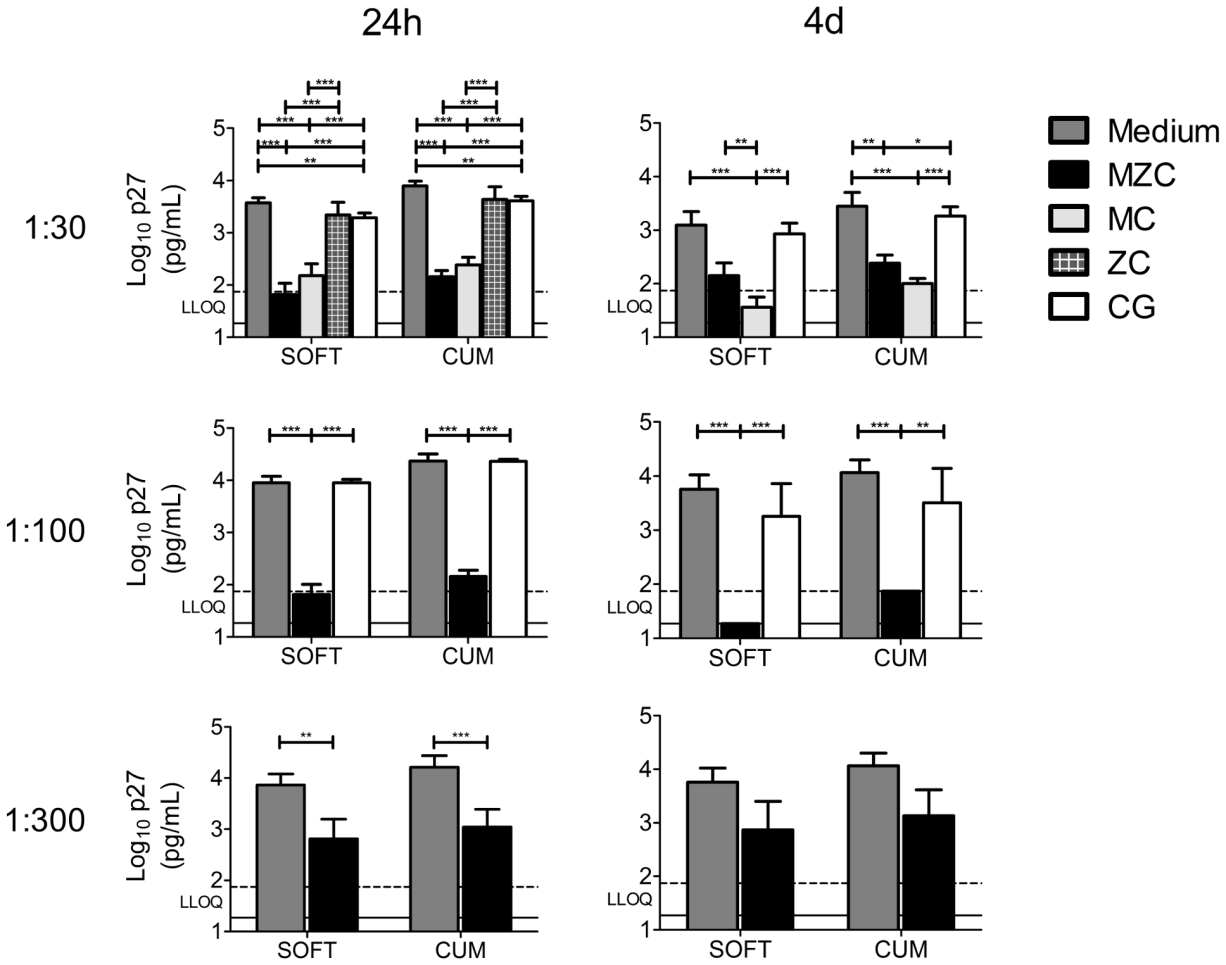
MIV-150 containing gels inhibit SHIV-RT infection in macaque vaginal explants up to 4d post gel exposure. Explants were challenged with SHIV-RT 24 h or 4d after exposure to diluted modified gels and cultured for 14d as described in Fig. 3. Shown are log_10_-transformed p27 SOFT and CUM analyses (Mean±SEM) (d3-14 of culture). Summaries of n = 6–16 (24 h post gel exposure) and n = 3–6 (4d post gel exposure) experiments are shown. The LLOQ of the assay are denoted for both SOFT (solid line) and CUM (dotted line).

Macaque vaginal explants from untreated animals were immersed in 1∶30–1∶300 diluted modified gels for ∼18 h in the presence of PHA/IL-2, then washed and challenged with 10^4^ TCID_50_ of SHIV-RT 24 h or 4d later, to quantify residual antiviral activity post gel washout. MZC strongly inhibited infection at 1∶30 (significant for CUM/SOFT in 24 h experiments; significant for CUM but not for SOFT in 4d experiments) and at 1∶100 (significant for CUM/SOFT) dilutions (Fig. 4). Comparable protection with 1∶30 and 1∶100 of MZC suggests that the inhibitory effect may be saturable. 1∶30 diluted MC (the only dilution tested) also significantly inhibited tissue infection (Fig. 4). The protection provided by both MZC and MC was significant compared to untreated infection controls as well as to CG placebo controls. A 1∶300 MZC dilution afforded less pronounced, but still significant, protection when tissues were challenged 24 h, but not 4d post gel exposure (Fig. 4). Exposure to CG significantly inhibited infection relative to the medium control in 24 h experiments (Fig. 4; 1∶30 gel dilution). ZC demonstrated no activity against SHIV-RT (Fig. 4). Additional experiments in which explants were transferred to new plates after gel exposure and washout determined that culture plate-bound MIV-150 does not significantly contribute to the observed MZC activity. The post washout activity of MZC against SHIV-RT challenge 24 h post gel exposure was not changed under these settings (n = 4 experiments; not shown).

**Figure 5:**
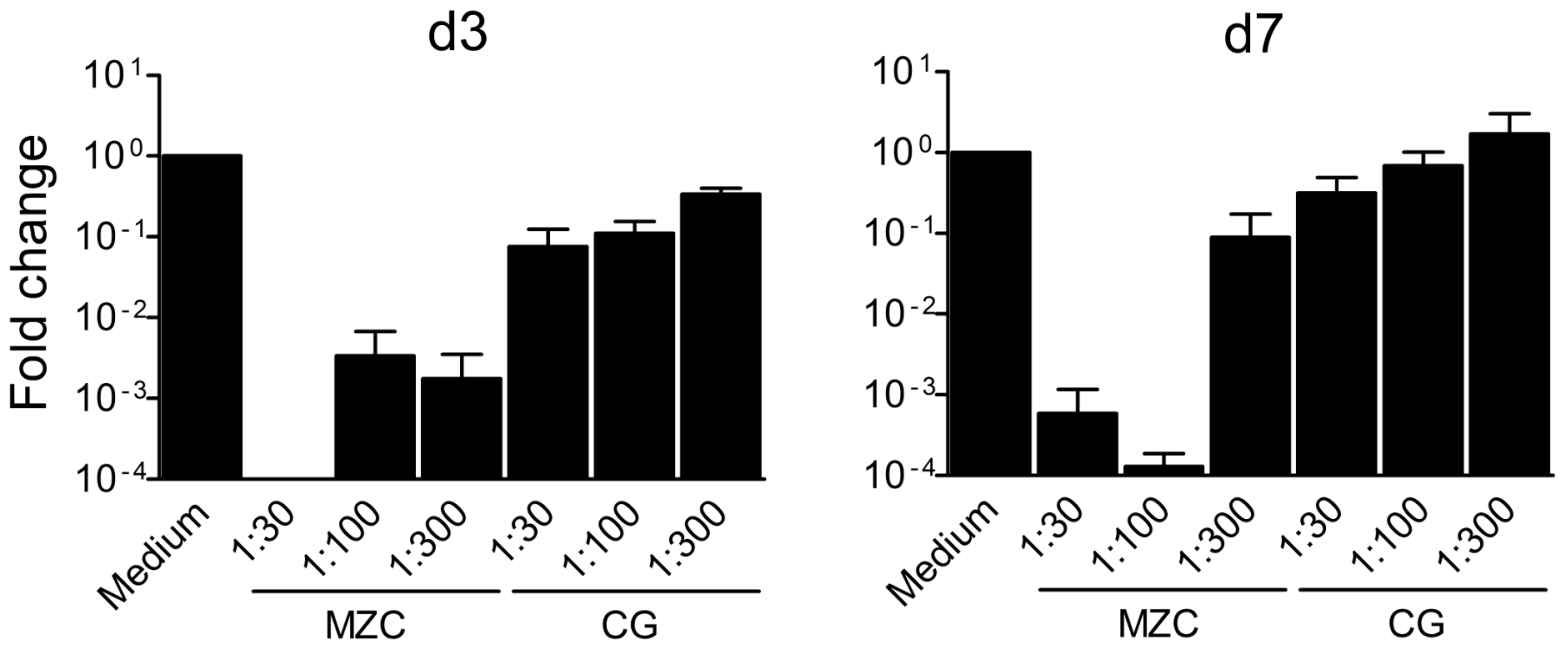
MZC inhibits SHIV-RT DNA burden in the tissues. Explants were challenged with SHIV-RT 24 h after exposure to diluted modified gels (Fig. 4). 3d (A) and 7d (B) after infection, tissues were collected and genomic DNA was extracted. Viral DNA was quantified by SIV *gag* qPCR. The 2^−ΔΔct^ method was used to calculate the fold change of SIV *gag* expression. Summary of n = 2 experiments at d3 and d7 each is shown (Mean±SEM).

SIV*gag* qPCR analysis using genomic DNA from tissues exposed to MZC gel (1∶30–1∶300 dilutions) and infected with SHIV-RT 24 h post gel exposure demonstrated inhibition of viral DNA (Fig. 5) and supported the ELISA results.

**Figure 6:**
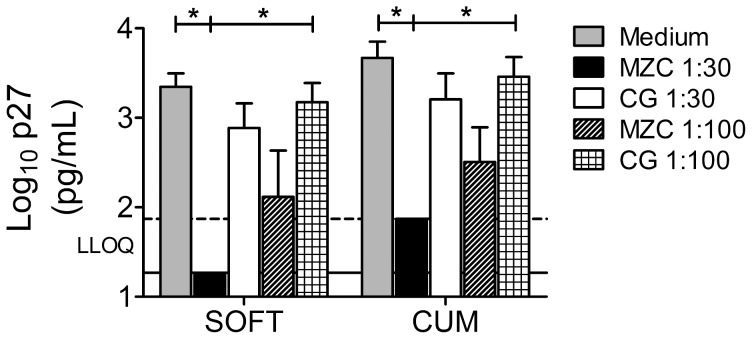
MZC inhibits SHIV-RT infection in macaque vaginal explants derived from Depo-treated animals. Vaginal explants from Depo-treated animals (5 wks post Depo) were challenged with SHIV-RT 24 h after exposure to diluted modified gels and cultured for 14d as described in Fig. 3. Shown are log_10_-transformed p27 SOFT and CUM analyses (Mean±SEM) (d3-14 of culture). Summary of n = 3–5 experiments is shown. The LLOQ of the assay are denoted for both SOFT (solid line) and CUM (dotted line).

To determine whether Depo treatment affects the *ex vivo* activity of MZC and to further relate *ex vivo* testing results with data from efficacy studies [2]–[4], vaginal biopsies from Depo-treated animals were exposed to MZC, washed, and challenged with SHIV-RT 24 h later. The activity of the gel at 1∶30 dilution was not changed; however, some decrease in the activity at 1∶100 dilution was observed providing 90/88% (CUM/SOFT) inhibition (Fig. 6) as compared to 99/99% (CUM/SOFT) inhibition in tissues from untreated animals (Fig. 4).

### Unformulated MIV-150 protects macaque vaginal explants against SHIV-RT infection

**Figure 7:**
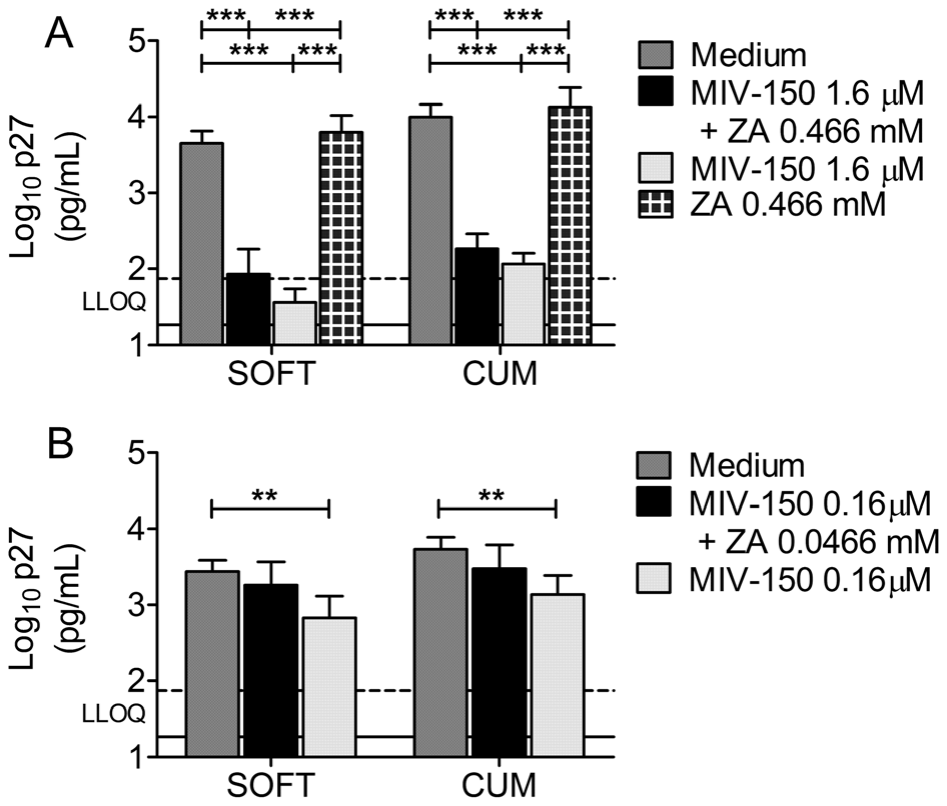
Unformulated MIV-150 inhibits SHIV-RT infection in macaque vaginal explants. PHA/IL-2 stimulated explants were challenged with SHIV-RT (10^4^ TCID_50_/explant; 2–4 explants/condition) 24 h after ∼18 h exposure to unformulated MIV-150 (1.6 or 0.16 µM), ZA (0.466 mM or 0.0466 mM), or a combination of both vs. untreated control (Medium) (A, B). After challenge, the tissues were washed and cultured for 14d in the presence of IL-2. Shown are log_10_-transformed p27 SOFT and CUM analyses (Mean±SEM) (d3-14 of culture). Summaries of n = 3–11 experiments are shown. The LLOQ of the assay are denoted for both SOFT (solid line) and CUM (dotted line).

Although addition of ZA to the MC gel improved protection against SHIV-RT infection *in vivo* after repeated gel administration [2], our data suggest that the bulk of the antiviral activity measured herein is due to MIV-150. To explore this further, we tested the antiviral activity of unformulated MIV-150 and of a combination of unformulated MIV-150 and ZA (non-toxic concentrations; MTT analysis not shown). To mimic the approach used in the experiments with gels, macaque vaginal explants were incubated with 1.6 µM MIV-150 and 0.466 mM ZA (corresponding to 1∶30 gel dilution) for ∼18 h, then washed and challenged with SHIV-RT 24 h post APIs exposure. Exposure to MIV-150 or the combination of MIV-150 and ZA inhibited infection similarly to the corresponding MZC or MC gels (Fig. 7A). ZA did not inhibit SHIV-RT infection *ex vivo* (Fig. 7A). Decreasing MIV-150 to 0.16 µM resulted in less significant protection (Fig. 7B).

### *In vivo* application of MZC and CG leads to decreased *ex vivo* SHIV-RT infection in vaginal tissues

To determine if *in vivo* administration of MZC leads to inhibition of *ex vivo* SHIV-RT infection in the genital mucosa, macaques received vaginal application of MZC or CG daily for 14d, mimicking *in vivo* efficacy studies [2], [3]. 24 h after the last gel application, vaginal and ectocervical biopsies were collected and challenged *ex vivo* with SHIV-RT. The VF MIV-150 concentrations in MZC exposed animals ranged from undetectable to 7.9 nM. We examined infection levels in tissues collected both prior to and after the respective gel dosing for each animal.

**Figure 8:**
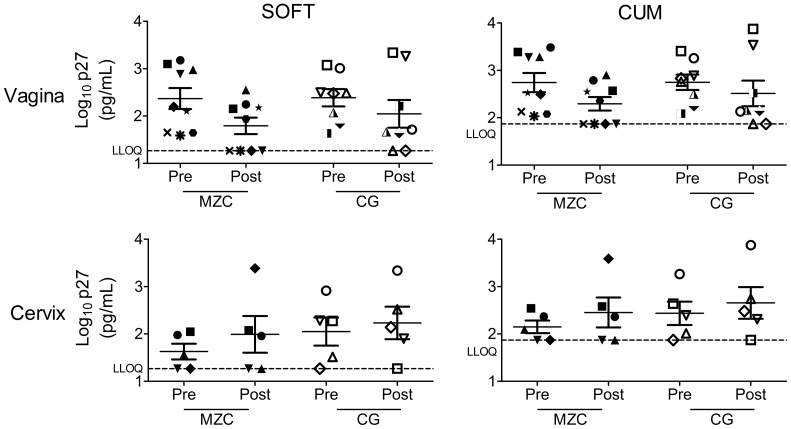
Repeated vaginal administration of MZC and CG reduce *ex vivo* infection of the vaginal mucosa. Depo-treated macaques were administered daily original or modified MZC vs. CG intravaginally for 2 wks. Vaginal biopsies were collected at the beginning of the study (baseline) and 24 h after the last gel application as described in Fig 1. Cervical biopsies were taken only from macaques treated with modified gels. Explants (3–6 vaginal and 2–5 cervical) were challenged with 10^5^ TCID_50_ SHIV-RT/explant in the presence of IL-2. After ∼18 h incubation, tissues were washed and cultured for 14d in the presence of IL-2. Shown are log_10_-transformed p27 SOFT and CUM analyses (Mean±SEM). Each symbol indicates an individual animal.

Infection levels in vaginal biopsies taken 24 h after the last gel were reduced (51–62% inhibition) by both MZC and CG gels relative to baselines (Fig. 8, Table 2), indicating a barrier effect of CG. Although reduced to a greater extent, the infection inhibition after MZC exposure (65–75%) was not significantly different from infection inhibition after CG exposure (32–45%). To increase the samples size, PD data on original formulations were included in these studies. There was no statistically significant difference in the inhibitory effect afforded by original and modified formulations. No inhibitory effect on *ex vivo* infection was observed in ectocervical biopsies collected 24 h after the MZC or CG exposure.

**Table 2:** PD study.

Predictor	SOFT				CUM			
Pairwise Comparison	Ratio	[95% CI]	p-value^1^		Ratio	[95% CI]	p-value^1^	
**Vaginal tissue only:**								
Time: Post Gel vs. Baseline	0.38	[0.18, 0.79]	**0.0114**	*	0.49	[0.26, 0.91]	**0.0249**	*
Interaction: Time – Gel (MZC, CG)		Overall	0.2929			Overall	0.2753	
Interaction: Time – Gel (original, modified)		Overall	0.5219			Overall	0.5538	
**Cervical tissue only** 2 **:**								
Time: Post Gel vs. Baseline	1.86	[0.50, 7.00]	0.6124		0.85	[0.43, 1.69]	0.6300	
Interaction: Time – Gel (MZC, CG)		Overall	0.9355			Overall	0.9393	

1 Included are the Tukey-Kramer adjusted 95% confidence interval (95% CI) and p value for multiple pairwise comparisons.

Significant p values <0.05 (*) are indicated in boldface.

2 Modified gels only.

## Discussion

We demonstrate that MZC is safe in the macaque and human genital mucosal models based on histological evaluation and MTT assay. These results support previous data showing that MZC is safe *in vitro* and *in vivo* (mice and macaques) [2], [3].

When present at the time of *ex vivo* viral challenge, the gel provides strong protection against SHIV-RT in macaque vaginal mucosa up to 1∶300 dilution, indicating that its activity is unlikely to be affected by dilution with VF or dilution associated with intercourse. If gel (2–4 ml) and VF (∼ 0.5 ml) [24] mix, the net dilution effect is estimated to be 10%–30% [25], [26]. In addition, there will be a range of dilutions of a gel by semen over time after ejaculation. However, given that the volume of the human ejaculate ranges from about 2 to 5 mL [24], these dilutions will be well below the 1∶300 dilution that has been shown to be efficacious in the current study. A single *ex vivo* exposure to 1∶30 and 1∶100 diluted gel 24 h or 4d prior to virus exposure afforded significant inhibition of infection. Notably, the study design (different animals included in different experimental sets, small sample size) did not allow comparison of tissue susceptibility to SHIV-RT at varying *ex vivo* challenge time-points, to determine if changes in susceptibility to infection contributed to the observed results.

As previously reported for neat MIV-150 in cell-based assays [27], the antiviral activity of MZC in the tissues was not changed in the presence of SP. It has also been shown that VF and semen does not interfere with the activity of *in vivo* intravaginal ring (IVR)-released MIV-150 in TZMbl cells [13](Kenney et al., in preparation; Ugaonkar et al., in preparation) and in macaque vaginal explants *ex vivo* [17].

MZC was reported to be more potent than MC against SHIV-RT *in vivo* [2]. To start to address the potential mechanism of the improved antiviral activity, we compared the activity of combined unformulated MIV-150 and ZA to that of either compound alone. The combination of MIV-150 and ZA afforded no greater anti-viral activity compared to MIV-150 alone. Thus, the antiviral activity measured in this system was dominated by MIV-150. Given the immunomodulatory properties of zinc [7]–[10] and the fact that repeated administration of ZC showed greater efficacy in macaques than a single dose [2], [4], [5], ZA can potentially create a non-permissive environment for viral transmission that requires more than the single exposure to ZA as utilized herein. Our observation demonstrating no antiviral activity of a single exposure to ZC in explants supports these data.

We recently demonstrated in a proof-of-principle study that tissue-associated MIV-150 released *in vivo* post MIV-150 IVR exposure inhibits *ex vivo* SHIV-RT infection [17]. Repeated *in vivo* MZC and CG dosing provided 51–62% inhibition (significant) of *ex vivo* SHIV-RT infection in vaginal mucosa 24 h post last gel exposure, indicating a barrier effect of CG. Although the effect of MZC did not significantly differ from CG, MZC afforded 65–74% inhibition of infection level vs. 32–45% inhibition afforded by CG. Because of the barrier effect of CG, we are unable to determine if there would be more antiviral activity 8 h after gel exposure, the time at which consistent complete protection against vaginal SHIV-RT challenge was observed *in vivo* [2], [3]. It is plausible that MZC activity in PD studies could have been stronger at the earlier time-point as tissue MIV-150 concentrations were reported to be higher 8 h after last MZC exposure than at 24 h [2]. The barrier effect of CG in PD studies and in some experiments where tissues were immersed in diluted gel and challenged *ex vivo* is in contrast to *in vivo* efficacy data [2], [3]. We cannot exclude that incomplete washout of the gel from biopsies could have contributed to this effect.

Higher protection against *ex vivo* infection was observed when tissues were immersed in diluted MZC vs. *in vivo* gel application. This could be due to greater gel/API access to mucosa and submucosa in *ex vivo* gel exposure experiments vs. exposure limited to the epithelial side during *in vivo* gel application. Some decrease in MZC *ex vivo* activity at higher gel dilution (1∶100) was observed in tissues from Depo-treated animals providing 88–90% infection inhibition as compared to 99% inhibition in tissues from non-Depo-treated animals. This could have also contributed to PD results as Depo-treated animals were utilized for PD studies. Our results and published data [28], [29] emphasize the need to study the impact of hormonal contraceptives on mucosal HIV infection. Furthermore, Depo treatment was reported to change biodistribution of HIV inhibitors leading to decreased tissue concentrations and increased plasma levels [30].

Although the amounts of MIV-150 in the ectocervix and vagina 24 h post last gel were reported to be similar [2], no trend of decreased infection and no barrier effect of CG in the ectocervical mucosa were detected in our study. This finding warrants further investigation. Post-Depo treatment infection level in the cervix in the placebo group was higher than the infection at the baseline, although this was not statistically significant. Changes in cervical tissue susceptibility to infection post-Depo treatment could have potentially contributed to the lack of MZC activity in the cervix. However, the sample size in the study was too small to determine the difference. We need to note that we were unable to measure tissue levels of MIV-150 in our study and had to extrapolate data from earlier study [2].

As discussed in [22], [23], there is currently no consensus on the optimal virological endpoint for characterizing tissue infection. PK/PD analysis of UC-781 gel in a Phase 1 rectal safety study [22] demonstrated that SOFT and CUM measurements at the higher titer of HIV_BaL_ (10^4^ TCID_50_) had reliability and provided evidence of between-visit reproducibility. SOFT analysis defines an endpoint at which high rates of virus growth have been achieved and no further biologically significant increases in virus growth are apparent [22]. It is less affected by the duration of the explant assay than CUM analysis. Inclusion of SOFT analyses is important for potential comparison of data across laboratories using longer explant assay protocols [22], [23]. Based on this, we performed both analyses in our study.

In conclusion, our data show that MZC is safe in macaque vaginal and human ectocervical mucosa. The gel is highly effective against *ex vivo* SHIV-RT infection in macaque vaginal explants (gel/virus co-exposure experiments and post gel washout effect). Although in our PD studies MZC activity (65–74% inhibition) 24 h post last gel administration did not significantly differ from CG (32–45% inhibition), it was comparable to the protection seen when macaques were vaginally challenged 24 h after gel administration (∼75%) [2]–[4]. Overall, the data suggest that evaluation of safety, *ex vivo* activity and PK and PD in macaque explants can inform macaque efficacy and clinical studies design, and support advancing MZC for clinical evaluation.
